# Supplementary material for: Resilience throughout COVID-19: Unmasking the realities of COVID-19 and vaccination facilitators, barriers, and attitudes among Black Canadians
Source: PLoS One. 2024 Aug 20;19(8):e0304904. doi: 10.1371/journal.pone.0304904 (PMC11335130; doi:10.1371/journal.pone.0304904)
Supplement: S1 Data — (DOCX) [file pone.0304904.s001.docx]

# Semi-structured Interview Questions

Impact of COVID-19 on Black communities

- How has COVID-19 impacted the quality of your life?
- What are the COVID-19 public health restrictions you know of?
  - How have these COVID-19-related public health restrictions impacted you?

Attitudes and experiences towards COVID19 vaccines

- How do you feel about the COVID-19 vaccine?
- Where do you get your information on vaccines for COVID-19?

Recovery from COVID19

- Have you or anyone close to you ever tested positive for COVID-19?
  - If so, how was your experience in your recovery?
  - How did you get support (from family or friends) during your recovery?
  - Did you experience stigma during or after being infected with COVID?

# Demographic Questions

1. What is your ethnicity?
2. What is your gender?
3. What is the city, and the first three characters of the Zip code you live in, and how long have you lived here?
4. What is your age?
5. What is your marital status?
6. What is your current employment status?
7. What is the highest degree or level of school you have completed?
