# Supplementary material for: Resilience throughout COVID-19: Unmasking the realities of COVID-19 and vaccination facilitators, barriers, and attitudes among Black Canadians
Source: PLoS One. 2024 Aug 20;19(8):e0304904. doi: 10.1371/journal.pone.0304904 (PMC11335130; doi:10.1371/journal.pone.0304904)
Supplement: S1 Table — (DOCX) [file pone.0304904.s002.docx]

***S1 Table: Codes and their representative quotes related to Experience with COVID-19, Impact of COVID-19, and Experience with COVID-19 Vaccines* (n = 134)***

| **Experience with COVID** | | | |
| --- | --- | --- | --- |
| **Code name** | **Quote** | **Participant ID** | **Number of participants** |
| Family member(s) got COVID | “I had family in the UK, very serious. Family in Zimbabwe, very seriously ill. Family in South Africa, very seriously ill.” [Participant ID_132] | 003, 007, 022, 063, 073, 085, 086, 089, 105, 109, 121, 132 | 12 |
| Family member(s) hospitalized | “My father, he was affected early, April 2020, and he was at the hospital for 11 days.” [Participant ID_032] | 003, 007, 015, 032, 073, 105 | 6 |
| Family member(s) passed away due to COVID | “We had five deaths. My daughter and her husband had five deaths, including her father-in-law, and her sister-in-law, and their aunt.” [Participant ID_132] | 007, 023, 073, 105, 118, 128, 132, Focus group (QC) | 8 |
| Family or friends provided support | “In my friend group we supported each other and narrowed our covid circle to just us + close family in late 2021.” | Focus group (QC, AB) | 2 |
| Felt guilty for getting COVID | “One of my male cousins felt some internalised guilt and internalised shame because he blamed himself for potentially bringing back COVID to his household bubble and potentially infecting one of my female cousins and one of my aunts.” | Focus group (ON) | 1 |
| Friend(s) hospitalized | “So he said it was really bad, and he was hospitalized in the hospital for a few days.” [Participant ID_027] | 027 | 1 |
| Hospitalized due to severity, required oxygen | “So yes, I’ve had relatives test positive for COVID. No one has passed away in my family tree thus far, thank goodness, but some folks have ended up hospitalised or with severe COVID symptoms in quarantine or isolation.” | Focus group (ON) | 1 |
| Someone close died | “Their parents caught it, and both parents died” [Participant ID_049] | 027, 049, 078 | 3 |
| Someone close got hospitalized | “The dad had to be admitted in the hospital and all of that.” [Participant ID_115] | 078, 115 | 2 |
| Difficult for some days | “First couple days were difficult.” [Participant ID_062] | 062, 096, 109, 125 | 4 |
| Fine after a few days | “I was able to recover after a few days” [Participant ID_081] | 034, 062, 081, 117, 118, 120 | 6 |
| Long recovery | “However, I do know of people who had much longer recover times based on underlying health issues.” [Participant ID_094] | 020, 065, 083, 094, 101 | 5 |
| Over 2 weeks to recover | “His recovery took time, and we were really worried for him because of his age, and he was very sick. His recovery took almost two week sto one month. [Participant ID_032] | 030, 032, 045, 046, 048, 054, 103, 105, Focus group (QC) | 9 |
| Over 4 weeks before testing negative | “It was really hard to get tested negative after I contracted it. I spent a 37 days before I test negative, because if you have to travel then you have to test negative.” [Participant ID_106] | 106, 128 | 2 |
| Recovered quickly | “Well, let's say the recovery - I recovered a little faster than I thought I would.” [Participant ID_081] | 081, 093, 094, 117, 119, 120 | 6 |
| Recovered within 2 weeks | “And I think their recovery was within the two weeks period.” [Participant ID_125] | 011, 032, 049, 053, 054, 059, 060, 063, 075, 077, 078, 101, 102, 117, 118, 125, 126, Focus group (AB, QC) | 19 |
| Required oxygen therapy | “Not able to breathe without a ventilator.”[Participant ID_015] | 003, 007, 015 | 3 |
| Scary experience | “Scary in that it was definitely a very intense version of what I feel may relate to the flu.” [Participant ID_097] | 097 | 1 |
| Severe symptoms and difficult recovery | “And I do know some people who said that they thought they were going to die and they didn’t believe they were going to be able to recover from this.” [Participant ID_107] | 003, 068, 078, 082, 099, 103, 105, 107, 109, 110, 118 | 11 |
| Slow recovery | “The recovery was very slow.” [Participant ID_065] | 065 | 1 |
| Stayed in ICU | “The one was I think was in the ICU for a while and it took a long time for that person to recover and recovery is not one dimensional.” [Participant ID_020] | 020, 105 | 2 |
| Experienced stigma | “But we contracted in the early days of the pandemic and I felt like we had leprosy and people kept their distance. And I think that I attribute that to just how it was viewed that it was only certain groups, whether you were homeless or poor or racialized. So, it was like a disease that affected certain groups. Now that it's affecting everybody the stigma is not as much there..” [Participant ID_059] | 007, 022, 023, 024, 026, 027, 048, 059, 100, 110, 129, 131, 132 | 13 |
| Family member ostracized | “My son works at a vaccination clinic in Mississauga, Ontario, and he got COVID, and he brought it into the house with his father, his father’s wife, and her son, and they were very angry because they’re not vaccinated, they’ve chosen not to get vaccinated, and it’s caused major arguments and a rift in that household. My son was ostracised by them for getting the vaccine and for working in the clinic, because they don’t believe in any of that.” [Participant ID_023] | 023 | 1 |
| Family members experienced stigma | “I think they did, because I know that for family members, not immediate family, but part of wider family network WhatsApp, almost in jest. But now that you say that, maybe, actually I have no idea what they felt, maybe they felt stigma within that. Now that you’ve said that, maybe it’s something they thought about, but not saying” [Particpant ID_076] | 064, 076, 131 | 3 |
| Someone close got stigmatized | “Yes, I would say that she did, because even I was like, she’s coming back. How are we going to react around her? Not that she was coming back, but she’s coming to join us, and we know that she’s just had COVID.” [Participant ID_115] | 022, 040, 074, 076, 115 | 5 |
| Stigma for getting COVID after not getting vaccine | “Possibly, I couldn’t say for sure because they were adamant on not being vaccinated and then they were found positive, I think that was maybe – I’m sure people had mentioned to them that that could have been avoided if they had taken the vaccine when it was offered.” [Participant ID_011] | 011 | 1 |
| Didn’t need support | “There was really no support needed.” [Participant ID_022] | 022, 034 | 2 |
| Family lacked support | “No, they didn't get any support.” [Participant ID_063] | 034, 063 | 2 |
| Inadequate financial support due to employment status | “Especially financially, I know that’s the hardest thing for them. Because some were not fully employed, some were casual employees or things like that, they don’t get full benefits.” [Participants ID_029] | 029, 072 | 2 |
| Lack financial support due to immigration status | “And some are immigrants so they definitely do not get the benefits, although people are getting CERB regardless of status.” [Participant ID_029] | 029 | 1 |
| Lacked informational support | “There was no information, there was no set up yet for it. There was no support.” [Participant ID_100] | 100 | 1 |
| Lacked support | “So yes, we didn't really have support because everybody was sick around us.” [Participant ID_106] | 052, 059, 083, 106, 111, 118 | 6 |
| Person didn’t get support | “No, they didn't get any support from any other people.” [Participant ID_063] | 063 | 1 |
| **Impact of COVID on** | | | |
| **Code name** | **Quote** | **Participant ID** | **Number of participants** |
| Added stressors | “So they had that, and then the uncertainty of the pandemic, and there were just added stressors there that people were already dealing with.” [Participant ID_064] | 003, 064, 089 | 3 |
| Adjusting to work-at-home life | “I changed my routine. I would wake up in the morning, read, then get ready for work, start working at my desk.” [Participant ID_115] | 016, 020, 025, 057, 067, 074, 078, 099, 115 | 9 |
| Altered interaction with students | “Normally I’m the type that like to relate well with my student. You meet them on the hallway, or you give them a high five at least. You cannot do that again. So you cannot touch them, you cannot give them high five and they cannot come close to you.” [Participant ID_027] | 027 | 1 |
| Altered mode of transportation | “That’s my strong preference, is to use public transportation rather than driving my own vehicle, just from a climate impact perspective. But I don’t have any choice now, I have to drive my vehicle, and really have had to avoid community events.” [Participant ID_046] | 046 | 1 |
| Altered routine and activities | “It's something, honestly, that changed our life and routine activity, so, yes, it’s affecting us. It’s affected us.” [Participant ID_032] | 032, 052, 060, 117 | 4 |
| Business was lost | “And then also my business is actually, the [unclear], is actually dissolving because we couldn't agree on our COVID safety measures and vaccination protocols. So yes, we just weren't on the same page. So, unfortunately I lost the business as a result as well.” [Participant ID_062] | 062 | 1 |
| Challenges adjusting to remote work or school | “But I would say the COVID helped a lot of families put things back together because you have to spend more time. And some jobs transition from being physical to online, that’s practising the hybrid and remote kind of jobs. For those working from home, yes, it wasn't easy trying to adjust your lifestyle to doing things from home and then separating work from house chores. But I think it gave more quality time with family members, from my perspective. I feel the COVID has to an extent helped many homes, made relationships better.” | Focus group (BC, ON) | 2 |
| Challenging as a single parent | “You don't have anyone at home to watch them? It's one person per family. No, because I'm a single mother and that's reality.” [Participant ID_052] | 052, Focus group (QC) | 2 |
| Change in job structure | “I think my job is [pause] because of the way that it is structured. There are changes that are being made that are beneficial for some but not beneficial for everyone.” [Participant ID_011] | 011, 012, 016, 024, 025, 026 | 6 |
| Concerned about access to basic needs | “What can I say? I think at the beginning of the pandemic there was a lot of uncertainty, a lot of people were panicking and worrying, so access to basic needs and this kind of thing.” [Participant ID_064] | 064 | 1 |
| Concerned about getting COVID | “You ask yourself “If I get the COVID, am I gonna survive or am I gonna die?’” [Participant ID_006] | 006, 017, 023, 027, 031, 065, 090, 103 | 8 |
| Concerned for others wellbeing | “Early in COVID – with COVID 19, I was very concerned for family members who lived in the United states and we’re very very sick with COVID.” [Participant ID_015] | 015, 031, 095, 120, | 4 |
| Confined to home | “Over the summer, especially, not everybody has air conditioning. So you're confined in a home. And so that's difficult.” [Participant ID_112] | 112 | 1 |
| Created community divide | “And I think that the mandates that have come with it as well, have created divided opinions…So I think COVID-19 has just created a lot of compounding uncertainty within community. And made it harder for communities to connect.” [Participant ID_ 111] | 111 | 1 |
| Decreased physical activity | “The gyms were closed. So, it was little bit harder to find things to do, to move. And everybody was working out outside. And, yes, there were just a lot of people outside because the gyms were closed. And, yes, people didn’t want to go grocery shopping because they were scared at first. So, it was a lot of ordering food in. And I think it just impacted my overall physical and mental health.” | Focus group (AB) | 1 |
| Decreased productivity | “Firstly it’s cut down on the productivity of the quality of the things that I normally do because of the social distance and constantly watching that I have to sanitise.” [Participant ID_113] | 113 | 1 |
| Difficulty adjusting post-pandemic | “I think its difficult adjusting to post pandemic. Fear of large gathering amongst many people.” | Focus group (BC) | 1 |
| Difficulty finding affordable housing | “The cost of living has now gone up. Because people are moving out to certain places, people are trying to live by themselves, I’m really struggling to find somewhere that I can afford to live in.” [Participant ID_084] | 084 | 1 |
| Difficulty getting a job | “Secondly, was also access to jobs was very, very challenging because most of companies shut down and trying to apply for jobs was very difficult to be hired or to get the job.” [Participant ID_082] | 038, 042, 081, 082, 084, 090, 091, 099, 100, 111, 119, 124, 128, Focus group (BC, ON) | 15 |
| Difficulty shopping with kids | “One of my biggest barriers was being the sole parent to my children, and having to bring them into stores with me, I had many incidents where I was judged and even yelled at.” | ￼ Focus group (QC) | 1 |
| Difficulty traveling | “I actually told him the issues surrounding travel, it’s just too complicated, just don’t bother.” [Participants ID_020] | 002, 013, 020, 049, 058, 100, 110, 123 | 8 |
| Difficulty working remotely with kids | “Then I think another challenge which I felt impacted my life then was because I had kids. I think a lot of people that were parents, being that the lockdown. everyone had to be home. It was difficult dealing with your kids at home and trying to handle a full-time job. There has to be the kid standoff, create some, let me say distraction to your work. You have to find a way to actually adjust to having these kids, trying to meet your target at work, trying to make sure your work is properly done. I think that was one of the major impacts for me.” | Focus group (BC) | 1 |
| Feels targeted by Government | “It’s the government and their policies and their mandates and their attack on my community that has affected me greatly. Being watched by the police more, seeing the negative press in the media, with them weaponizing, and making up, and fabricating terminology like vaccine hesitancy and then showing images of Black people constantly, when they’re using this weaponized terminology.” [Participant ID_010] | 010 | 1 |
| Felt lonely | “And a lot of us came to the realisation that a lot of us are alone, that unlike our white colleagues, we don't have generations of a family in Canada, in the city, to rely on for support, emotional support, to bring us food, to check in on us, etc., and how important friends become in your new family. I have a very small family, and so, I realised that with a lot of my white friends, it was different. Their grandmothers were checking in, they're great aunts. And also, another thing is that a lot of the colleagues that I have, they live in relatively small places with fewer bedrooms than a lot of the white colleagues have. So, socially isolating was very, very difficult.” | Focus group (QC) | 1 |
| Felt mentally at peace | “So, from a black perspective, I would say that was one significant one. It was just your mind was at peace somewhat. Despite the whole Black Lives Matter, George Floyd thing, but at least there was that little bit of peace for the two years.” | Focus group (QC) | 1 |
| Food insecurity | “A lot of people experienced income gaps and food insecurity throughout the pandemic, including myself and in my family. I think there was a lot of economic upheaval that was experienced and speaking for myself, that definitely has occurred in my household.” | Focus group (ON) | 1 |
| Found time to spend with family | “Asides from the negative, and from my perspective I'm not really the outgoing type, so I wouldn't say I missed so much like socialising. But I would say the COVID helped a lot of families put things back together because you have to spend more time.” | Focus group (BC, QC) | 2 |
| Increased cost to travel | “Last year I had to travel out of the country. I think it was actually more costly for me to travel because I had to have COVID tests done. Here it was free. But from back home, I had to get it paid. I think Canada wanted it done from a reputable institution and we had to pay for COVID tests. It did make traveling more costly. Now they've removed some of the restrictions.” [Participant ID_096] | 096, 132 | 2 |
| Decreased income | “There was a decrease in income. There was a period in the first wave and first lockdown of temporary furlough and unemployment.” | Focus group (ON, AB) | 2 |
| Increased anxiety | “Covid-19 pandemic had me feeling like I was on house arrest, there was a lot of fear mongering on news and social media, so that caused me a lot of anxiety. My oldest daughter passed away from respiratory illness in 2016, so seeing all the things on the news and social media had a very negative impact on me.” | Focus group (QC, ON, BC, AB) | 4 |
| Interfered with life plans | “I know that my finances are not where they would have been if it wasn't for the pandemic. I know my social life is not where it would be. And my professional life is not where it would be because everything has been affected by COVID-19. I don't think that there's any part of my life that has not been negatively affected by the pandemic.” | Focus group (BC) | 1 |
| Fewer microaggressions or inequities experienced | “Also, someone else had mentioned not having to deal with microaggressions at work. That's huge. Oh, my goodness, that’s huge, from a woman perspective, from a black woman perspective. That's absolutely huge in terms of the benefits to it.” | Focus group (QC) | 1 |
| Limited access to support network | “I would say that because I'm first generation Canadian, my parents were born in the Caribbean, and at the university where I work, I'm part of a lot of anti-racism groups, so we discuss these things a lot, especially during COVID, how it affected us. And a lot of us came to the realisation that a lot of us are alone, that unlike our white colleagues, we don't have generations of a family in Canada, in the city, to rely on for support, emotional support, to bring us food, to check in on us, etc., and how important friends become in your new family.” | Focus group (QC) | 1 |
| Limited conclusive info about COVID | “I want to say that being a black person, the beginning of COVID, there was this alternative truth going around that black people couldn't get COVID, so some people I know were taking false comfort in that idea at the beginning and that was troubling to me.” | Focus group (QC) | 1 |
| Limited interactions | “I think socially, by I guess reducing the amount of interaction, especially during the lockdown periods which yeah, that has been quite a noticeable impact.” [Participant ID_043] | 001, 017, 018, 019, 033, 035, 043, 044, 075, 077, 078, 082, 088, 105, 113, 121, 124 | 17 |
| Lost friends to suicide | “And within my friend group, thankfully, no one close, there were people who ended up committing suicide as a result.” | Focus group (QC) | 1 |
| Lost friendship | “What I was going to say is about what happened during the pandemic. I think, to some extent, I lost some friends in the sense that during the pandemic, I was overly sensitive to possibilities of contacting the disease, and I was trying to guard myself because of my age, because of my health situation.” | Focus group (ON) | 1 |
| Mourned loss on Zoom | “You know, I mentioned my friend dying. We had to watch her [unclear] on Zoom, and all that stuff, so that was, of course, hard.” [Participant ID_047] | 047 | 1 |
| Negatively affected mental health | “It is a struggle to maintain a positive mental health. It is a struggle. And so, it’s intentional reminding of why there is a struggle. But I’m not going to lie. Some days I’m in bed. Some days it’s hard. It’s a shadow. It’s that sadness. I won’t say depression. But there’s a sadness and an isolation that leads to a sense of uncertainty and never-ending cycle.” [Participant ID_048] | 002, 003, 013, 038, 041, 042, 048, 050, 063, 066, 072, 073, 074, 080, 081, 093, 103, 118, 123, 124, 128 | 21 |
| Received leisure time | “But I'm now able to break it up with gardening, with making some art, going for a walk, doing laundry, and your life is so much more efficient and you're not trying to jam pack everything into a weekend anymore, which has been transformative. It’s been literally transformative in terms of how we live our lives.” | Focus group (QC) | 1 |
| Received work opportunity | “But for me, workwise, I did have a lot of rolling layoffs, but I work in a field where they are layoffs, but there were way more than normal. But a lot of my community work offered me positions that were paid during the pandemic, and I was very blessed to be doing things that I love and helping people during isolation and quarantine and different things.” | Focus group (QC, BC) | 2 |
| Relief being around black community | “I wouldn't say this anywhere else, but it was just such a relief to be nowhere near white people, other than through video camera. Most of my friends are black, my partner is black, my family's mostly black. It was just so comforting. And I didn't even really think about it, until we were out for my sister's birthday in April. We were at a bar and we were surrounded by white folks. I think we were back to being one of four black people at a venue. And the three of us just started each other like, whoa, we haven't done this for two years. This is weird. And it took a bit of mental adjustment, where as before, that was just the norm. So, from a black perspective, I would say that was one significant one.” | Focus group (QC) | 1 |
| Remote learning negative outcomes | “I think one of the biggest impacts I found, because I'm a student and I live in a city my family where my family is not to study. So, the fact that that the shift to remote learning, as opposed to being able to go into campus, to socialise and engage, and just be around other students. I felt like that was a big loss.” | Focus group (QC) | 1 |
| Restricted mobility | “I said, it has affected our lives as the majority of the people because we are not as mobile as we normally are. We could not have gone away, or taken a vacation or anything because we tried to stay put.” [Participant ID_009] | 009, 025, 029, 030, 046, 050, 053, 072, 112, 130 | 10 |
| Restricted traveling | “And travel, I couldn't travel to go see family. I'm not Canadian. It was either to just be stuck here until things got better.” [Participant ID_122] | 025, 085, 088, 093, 109, 122, 132 | 7 |
| Socially isolated | “Lots of isolation, not a lot of opportunity for socialising or meeting people.” [Participant ID_041] | 004, 007, 011, 020, 021, 022, 026, 030, 034, 039, 040, 041, 042, 047, 048, 054, 055, 058, 062, 065, 068, 069, 071, 071, 073, 074, 075, 078, 079, 082, 087, 088, 096, 097, 100, 102, 103, 107, 110, 122, 125, 129, Focus group (BC, ON, AB) | 45 |
| Social skills regressed | “Quality of life has changed because there is more isolation. I feel more socially anxious and more socially awkward, and I’ve heard many of my peers and colleagues say this, that some people feel as though their social skills has actually regressed.” | Focus group (ON) | 1 |
| Stressed | “I think it's negatively affected my health to some degree just in terms of more stress. And also, some aspects of coping with that, just coping with the pressures that have just arisen throughout this whole pandemic experience.” [Participant ID_034] | 034, 040, 074, 121 | 4 |
| Stressed about in person classes | “So that was actually a big switch after doing two years of online school and then just returning back to in-class last week. That was quite stressful, just switching back.” [Participant ID_053] | 053 | 1 |
| Stopped going out | “I stopped going out as much.” [Participant ID_087] | 087 | 1 |
| Unable to afford internet to book vaccine | “I'm a strong believer in science, and we all realised that the same vaccines were being offered to people, regardless of their race. But what I did notice, and it had never crossed my mind, that when I had made an appointment online and I was waiting in line to get my vaccine, there were two families, separate families, and they were black, and the security guard told them to leave because they hadn't booked appointments. And they were trying to tell the security guard they didn't they didn't have internet access at home. And because of that, I saw them turned away and there were no exceptions made. It's like either you can afford the internet or you can't, and sometimes we take that for granted.” | Focus group (QC) | 1 |
| Unable to celebrate events | “I guess I'll talk again about my experience. I graduated university in June 2020. Graduating right after COVID was very difficult. I think when it came socially, I missed out on things like graduation and my family being here. I wasn't able to interact with my friends or celebrate.” | Focus group (BC) | 1 |
| Unable to go to church | “I used to attend church, so all of that, there is no longer access to church services. Although we were doing it online, but it doesn’t have the same touch as being in a social group with other people.” [Participant ID_093] | 001, 017, 069, 093, 096, 112 | 6 |
| Unable to have social gatherings | “I think also access to social gatherings.” [Participant ID_093] | 093 | 1 |
| Unable to travel | “It stopped us from traveling.” [Participant ID_031] | 004, 008, 012, 027, 031, 034, 036, 042, 050, 052, 059, 063, 093, 103, 111, 118, 119, 124, Focus group (QC, AB) | 20 |
| Unable to visit family or friends | “I wanted to go back to Africa I think for three, four years now I have not been able to travel because of restrictions in traveling. I have a mother who’s over 90, I can’t go to visit her, I can’t go to see her” [Participant ID_027] | 001, 004, 008, 012, 017, 020, 027, 030, 032, 043, 046, 050, 054, 067, 074, 077, 081, 091, 092, 098, 100, 105, 110, 120, 122, 124, 130, 132, Focus group (BC, ON) | 33 |
| Working remotely increased convenience | “I would say that COVID positively impacted my life in the sense that I was able to work from home. I was able to focus more. Just avoid having to travel back and forth to work, to get dressed to be in meetings. In my workplace, you often sense inequity, you sense it, you feel it in your body, and you just you live it. And I felt that that was minimised when we were able to work from home and just have Zoom meetings. You didn't have to physically be in that space.” | Focus group (QC) | 1 |
| Worried about community | “And maybe people feel that maybe those folks who are bearing all of that grief are finally in a place where they can let it out. So, on that, I would say, I'm a little worried about our community going forward.” | Focus group (QC) | 1 |

| **Experience with COVID-19 Vaccines** | | | |
| --- | --- | --- | --- |
| **Code name** | **Quote** | **Participant ID** | **Number of participants** |
| Believes it's a tool to depopulate | “Clearly, it’s horseshit. It’s an attempt by the globalist tyrants, the World Economic Forum, etc., etc., etc. It’s not even a mystery. It’s all documented. It’s an attempt to depopulate. Not attempt. It’s a successful attempt.” [Participant ID_037] | 037 | 1 |
| Believes it's an experiment | “With COVID, it just seems like an experiment for science.” [Participant ID_090] | 090 | 1 |
| Believes its controlling and harmful | “It is a tool of control. And it is killing people.” [Participant ID_010] | 010, 033, 118 | 3 |
| Believes it's horrible | “It's horrible.” [Participant ID_105] | 033, 105 | 2 |
| Believes its necessary evil | “I think it’s a necessary evil. I have gotten the vaccine. I have some worries or some concerns about it, but the reality that people have died from it or that it can cause you a lot of distress and side-effects, has driven me to be more receptive to having the two vaccines. I’m planning to get the booster.” [Participant ID_040] | 040 | 1 |
| Believes it's not a solution | “I don’t think it’s perfect and I don’t think it’s a solution but it’s all we have right now. I responded to the vaccine mandate and I’m triple vaccinated.” [Participant ID_073] | 022, 073 | 2 |
| Believes unvaccinated are brave | “And it's irreversible at this point. Because some people that were brave enough or at least they were able to stand their ground, they didn't get vaccinated. And for some reason, they might still be fine after all.” | Focus group (BC) | 1 |
| Believes vaccine is useless | “So once again, that is an unnecessary medical procedure that is being forced on people. It is not effective; it is not necessary.” [Participant ID_010] | 010, 022, 063, 072 | 4 |
| Believes vaccine protects the vulnerable | “I think, personally, what made me decide to get vaccinated was my mum. As I’ve said before, she’s a senior and she lives on her own. She was in our bubble. I was very apprehensive at first about it, but participated in many Zooms where I listened and took the information in and made a decision that I’m going to do this.” | Focus group (ON) | 1 |
| Comfortable with vaccine | “Yes, I can speak on my experience and attitudes. Generally, I've been someone who's very pro-vaccine. I've had to get vaccines my entire life. I've never had an issue with them. The idea of a COVID-19 vaccine in and of itself was not a thing that I had any reservations towards. I had a lot of family members and close family friends who maybe had backgrounds in science or had been keeping up with the research. And I trusted their thoughts and opinions on the vaccine. I did not feel any reservations towards myself getting it.” | Focus group (AB, BC, ON, QC) | 4 |
| Disfavor of coercive tactics | “I guess the coercion into the workplace is either you lose your job or get the vaccine. I didn't really appreciate that approach when there's not enough information out there.” [Participant ID_008] | 008, 033, 049, 053, 059, 065, 118, 120, 124, 125 | 10 |
| Dislikes vaccine being politicized | “I strongly dislike how it’s been politicized. It almost represents freedom versus stupidity, depending on who’s looking at it, and so that’s how it’s been portrayed and how the narrative around who’s taking the vaccine and who’s not and for what reasons seems to be now. It’s unfortunate. I really dislike about the vaccines how people, they’ve been put into a position to have to make a choice about something that’s medical based on factors that are not medical, that are more political.” [Participant ID_034] | 034 | 1 |
| Doesn’t trust it | “I don’t agree with it. I was actually pressured at work, to get the vaccine, which I’m against. So, yes. I’m against it. I don’t know too much about it. I don’t have enough information to make the decision to get it willingly.” [Participant ID_133] | 107, 109, 133 | 3 |
| Fear due to uncertainty about COVID | “I think I echo what everyone else is saying, even though I wouldn't say I 100% trusted the vaccine, it's just that COVID scared me more than the vaccine, so that was the reason that I got it.” | Focus group (BC, QC) | 2 |
| Fearful of long-term impact | “But I guess maybe because of cases that Johnson & Johnson had had in the past with some of their products. There was this fear that, okay, their vaccine might have an impact later on or issues might come up. I think that is one of the attitudes a lot of people had towards it.” | Focus group (BC) | 1 |
| Feels its rushed | “I do agree that at some point I did feel like the whole process of getting the vaccine was rushed.” [Participant ID_105] | 105, 107 | 2 |
| Feels racially targeted upon vaccine passport request | “I've had more issues with the vaccine certificate/passport system in terms of experiencing anti-Black racism when establishments single me out to show my papers proving vaccination when I'm the only black person in the store.” | Focus group (ON) | 1 |
| Feels restricted from getting vaccine due to age | “The only issue was the fact that I'm younger so I had to wait a lot longer even though I was doing frontline work.” | Focus group (BC) | 1 |
| Feels the Government could be compassionate | “In the Caribbean, some folks are a little bit more apprehensive about taking vaccines. And there's some history with that and being rightfully fearful of doctors. I just felt like some of those conversations could have been more compassionate, from a government standpoint, in terms of talking and opening up discussions that been people against each other.” | Focus group (QC) | 1 |
| Polarized opinions in Black community | “And that stress, that pressure, led to other fractures in their lives and in the relationships between us, which was definitely negative. The fact that people have different opinions and different perspectives made you really want to walk on eggshells around the whole issue, even though it was such a primary issue going on in the world, which was a strain in general, communicating with your loved ones that you want to communicate with, because of the distance that was imposed by the pandemic. So, that was a very strange thing to deal with mentally.” | Focus group (QC) | 1 |
| Felt forced to get vaccine | “I felt like I was being forced and that I wasn't being given enough information or enough time to make an informed decision.” [Participant ID_041] | 033, 041, 090, 092, 106 | 5 |
| Lack of accommodation | “But what I did notice, and it had never crossed my mind, that when I had made an appointment online and I was waiting in line to get my vaccine, there were two families, separate families, and they were black, and the security guard told them to leave because they hadn't booked appointments. And they were trying to tell the security guard they didn't they didn't have internet access at home. And because of that, I saw them turned away and there were no exceptions made. It's like either you can afford the internet or you can't, and sometimes we take that for granted.” | Focus group (QC) | 1 |
| Lacked access to desired vaccine | “Appointments for the initial two doses were without any issues. But for the booster, there was confusion on the part of government I guess.” | Focus group (AB) | 1 |
| Misinformed or confused about vaccine | “My experience with the vaccine. I was fine taking it. It wasn't problematic for me. But the lead up to it was somewhat problematic and everything that surrounded it. I remember at the time, because I'm a relatively young person, and I remember at the time speaking with my family doctor, when I was actually able to make an appointment, and being told by the doctor that there was functionally no difference between these different varieties of vaccines and whatnot. But then, lo and behold, later on, it turns out that there were certain risks of certain heart conditions for younger men, specifically, that differed between some of these vaccines. So, I’d definitely say that the experience of not being well informed by my medical practitioner was a negative.” | Focus group (AB, QC) | 2 |
| Regrets taking vaccine | “Because some of my friends in my circles did not get vaccinated. And slowly by slowly we are moving towards a place where there will be no mandate potentially in the future. So then for me, in my mind, I'm like, oh, we are in different worlds and I'm not sure if I made the right decision. There's a bit of regret.” | Focus group (BC) | 1 |
| Reserved about vaccine | “Sometimes when I fell sick with COVID, I felt, oh, maybe the vaccine was good. But I still have reservations.” | Focus group (BC) | 1 |
| Skeptical | “I think at the beginning, I was sceptical of if they've done enough research.” [Participant ID_041] | 022, 041, 058, 072, 076, 082, 095, 107, 129 | 9 |
| Stressed about taking vaccine to keep employment | “I also found, not for me personally, but for people close to me in my life, I could see that the mandating of vaccines as a condition of their employment became a very stressful thing. A thing that seemed like it was taking personal autonomy away from them.” | Focus group (QC) | 1 |
| Vaccinated because it was required | “Personally, I don't know much about the vaccines. I don't think many people do. I'm not a scientist. But I needed a job and that's how I just ended up getting vaccinated.” | Focus group (BC, QC) | 2 |
| Vaccinated in secret due to stigma | “My experiences with the COVID vaccine have to do with how my family reacted to it. I’m the only person in my immediate family to get vaccinated, and the process of getting vaccinated was very difficult and very emotional. I had to do it in secret. I basically became a closeted vaccinated person because the COVID vaccines are heavily stigmatised in my family unit and family tree for many different reasons.” | Focus group (ON) | 1 |
| 2-dose vaccines provide more immunity | “I would say that, basically, I think when the vaccine came out, there was this notion that, okay, it's better you get two shots because Johnson & Johnson had one shot based on where I was then. I was in the US. There was this notion of, oh, why don't you go get two shots? You’ll have more immunity and things like that. I think that was the notion I had.” | Focus group (QC, BC) | 2 |
| Wary of constant vaccination | “So that’s probably my only apprehension, when it comes to COVID-19. I believe the vaccines work, but I’m wary of having to take them all of the time.” [Participant ID_068] | 022, 042, 068, 070, 101 | 5 |
| Would not get vaccines | “I just said, almost to get on the train where everyone else was going on. But if I had a choice, I wouldn't.” | Focus group (BC) | 1 |
| Inaccessibility to vaccine location | “And sometimes the ones that were available were really far, so you had to search until you found one that was really close. That was the only barrier.” [Participant ID_122] | 033, 038, 047, 060, 085, 094, 122 | 7 |
| Difficulty accessing vaccine | “Because there was a long queue of people with symptoms and all of that, so it wasn’t very accessible.” [Participant ID_085] | 065, 085 | 2 |
| Lack of access | “One of the challenges is accessibility. Accessing it.” [Participant ID_072] | 034, 072, 081, 083, 117, 128 | 6 |
| Lack of health coverage | “And also, just speaking for some of my friends as well who didn't have MCP cards. I don't know if you have MCP where you are. But here in our province, it's the public health insurance. If you don't have the card, like most international students who come here and some of them are black, some of them didn't really have the MCP cards and that prevented them from getting the vaccine at the time that they should have.” [Participant ID_117] | 117 | 1 |
| Lack of information | “It was, honestly, the lack of information. If it wasn't for people in the community posting where to get the vaccine or me having friends who were nurses and who were distributing vaccines at a centre, I probably wouldn't have known where to start and how to get a vaccine.” [Participant ID_050] | 042, 050, 097, 110, 112, 115 | 6 |
| Lack of knowledge | “Nobody took the time to educate us on, okay, we have the vaccine now.” [Participant ID_041] | 041, 064, 110, 112 | 4 |
| Lack of vaccine variety options | “And also another issue, I think having different vaccines would have also helped because some people couldn't go and get vaccinated because maybe they had one vaccine and they didn't have the other one.” [Participant ID_117] | 057, 117, 122 | 3 |
| Location decreased access | “I think it did take a lot longer than I would have preferred to secure an appointment, but that was based on, I think, my location. If I was willing to go outside of my immediate location, I likely would have been able to get my vaccination sooner.” [Participant 067] | 067, 094 | 2 |
| Long lines to get vaccine | “The queue because at first, the queue was so long. You would go out and spend a number of hours to be able to get the shot. So that was a major barrier for me.” [Participant ID_128] | 012, 022, 030, 048, 062, 086, 113, 126,128 | 9 |
| Misinformation or inconsistent information | “The only thing I would say is, at first, I wasn't sure which vaccine to take. There was a lot of information. There was a lot of misinformation so much that I may have delayed taking the vaccine so that I could get a specific vaccine.” [Participant ID_122] | 029, 032, 033, 034, 041, 058, 089, 094, 095, 107, 122, 125 | 12 |
| Unavailable for age group | “One of the challenges was the fact that the booking process was likely online and because the emphasis was always that you could go online and you could book, the option to book vaccines using other measures, whether it was by a phone call or a messaging system, were really not available to younger individuals.” [Participant ID_103] | 103 | 1 |
| Accessibility for those with a disability | “I would say thinking about people with disabilities. Not much thought I believe was given to how to accommodate people.” [Participant ID_059] | 059 | 1 |
| Better technology or booking system | “Technology that worked well, where there was this automatic, okay this is when you’re eligible, you know, it could send an email, this is where you can go, or here’s your appointment. Just something, just some of it could have been more automatic?” [Participant ID_064] | 039, 042, 046, 064, 105, 131 | 6 |
| Door-to-door vaccine service | “In Kenya, where I come from, they do door-to-door vaccines. And I think that would have been easier for me.” [Participant ID_100] | 100, 121, 128 | 3 |
| First-come-first-serve | “I think they should have just said, the vaccine is coming, let’s do it when it on a first-come, first-served basis.” [Participant ID_079] | 079 | 1 |
| Lack of information from government | “My issue is how the government disseminated information about how vaccines work and their efficiency. I don’t personally have an issue with the actual idea of getting vaccinated or having a vaccine. Just how the information was shared.” | 071, 105, 112 | 3 |
