# Supplementary material for: Resilience throughout COVID-19: Unmasking the realities of COVID-19 and vaccination facilitators, barriers, and attitudes among Black Canadians
Source: PLoS One. 2024 Aug 20;19(8):e0304904. doi: 10.1371/journal.pone.0304904 (PMC11335130; doi:10.1371/journal.pone.0304904)
Supplement: Attachment — Submitted filename: Reviewer Response_Dec 20.docx [file pone.0304904.s003.docx]

| **Reviewer #1:** | **Response** |
| --- | --- |
| Thank you for the wonderful qualitative research. I would like to suggest the following; | |
| - In methods, please try to discuss the major variables, how you have selected participants (slightly detail) and how did you maintain the consent. | - Thank you for your comment. We have the existing methods described in the manuscript: onvenience sampling through FBC mailing list and snowball sampling - Furthermore, we have also added the following sentences in the “data collection” section to include the consent process: - *The research team obtained informed consent from participants first by explaining the purpose of the project, as well as the roles, risks, and benefits of participation, and answering any questions or concerns. Individuals who agreed to participate in interviews signed the consent form and submitted it to the research team for filing before interviews commenced. Moreover, the research team re-iterated the voluntary nature of participation, and that participants had the choice to withdraw from the study at any point. Interviews were conducted virtually over Zoom and lasted approximately 45 minutes.* - *Similarly, individuals who agreed to participate in focus groups reviewed and signed the consent form. Before focus groups begun, the research team re-iterated the project’s aim, rationale, as well as the expected roles, risks and benefits of participation. Moreover, the research team notified participants that they have the choice to withdraw from the study at any point. Four focus groups, one for each province (Ontario, British Columbia, Alberta, and Quebec), were conducted, with four to seven participants in each group. Focus groups were held virtually over Zoom and lasted approximately 45 minutes.* |
| - In analysis, also mention how did you do quantitative analysis. | - Our study undertook a qualitative design, and all data generated came from interviews focus groups (including socio-demographic data). |
| - Also try to add the implication of the study for addressing the issues. | *We have integrated sentences that describe the implications of the study throughout the discussions section:*   - *Our study highlights the inadequacy of current health and social support systems for Black Canadians, worsening health equity disparities during pandemics. Policymakers must prioritize innovative, community-centric approaches, including substantial investments in mental health services (e.g., digital-based interventions to increase connection), housing, and employment support [8, 20].”* - *Another significant challenge lies in the small sample sizes of current studies, thereby complicating the generalization of findings to the broader population and necessitating the compilation of results from multiple studies to construct a comprehensive understanding of vaccine hesitancy across populations [20, 31]. Despite the absence of race-based data collection in Canada, clear evidence suggests worse health outcomes and higher vaccine hesitancy among Black Canadians, emphasizing the urgent need for such data to inform timely public health responses [20]. Tailoring strategies involving Black community members, health leaders, and academics is crucial for addressing disparities, while sustaining equitable COVID-19 response policies and prioritizing race-based data collection and analysis are essential steps for building a more inclusive and responsive public health system [20, 31].* |
| - Better to include the specific recommendations for improving the status | - Thank you for your comment. We have addressed this by including specific recommendations throughout the discussion section: - *Our study highlights the inadequacy of current health and social support systems for Black Canadians, worsening health equity disparities during pandemics. Policymakers must prioritize innovative, community-centric approaches, including substantial investments in mental health services (e.g., digital-based interventions to increase connection), housing, and employment support [8, 20].”* - *Another significant challenge lies in the small sample sizes of current studies, thereby complicating the generalization of findings to the broader population and necessitating the compilation of results from multiple studies to construct a comprehensive understanding of vaccine hesitancy across populations [20, 31]. Despite the absence of race-based data collection in Canada, clear evidence suggests worse health outcomes and higher vaccine hesitancy among Black Canadians, emphasizing the urgent need for such data to inform timely public health responses [20]. Tailoring strategies involving Black community members, health leaders, and academics is crucial for addressing disparities, while sustaining equitable COVID-19 response policies and prioritizing race-based data collection and analysis are essential steps for building a more inclusive and responsive public health system [20, 31].* |
| - Please check and correct the write-up errors | - We have made the following grammatical edits on lines 96, 101, 114, 116, 167, 171 (Ghanaian misspelt), and 400 |
| **Reviewer #2**: | |
|  |  |
| 1.Authors have mentioned the data collection process [line 107-118]. It is said that there were two instruments to collect the data- FGD and Semi structured Interview. Authors highlighted that they had informed verbally and they mentioned that there two written consent forms. There is little confusion that whether they have taken the informed consent. | - Thank you for your comment. We have added the following sentences in the “data collection” section to include the consent process: - *The research team obtained informed consent from participants first by explaining the purpose of the project, as well as the roles, risks, and benefits of participation, and answering any questions or concerns. Individuals who agreed to participate in interviews signed the consent form and submitted it to the research team for filing before interviews commenced. Moreover, the research team re-iterated the voluntary nature of participation, and that participants had the choice to withdraw from the study at any point. Interviews were conducted virtually over Zoom and lasted approximately 45 minutes.* - *Similarly, individuals who agreed to participate in focus groups reviewed and signed the consent form. Before focus groups begun, the research team re-iterated the project’s aim, rationale, as well as the expected roles, risks and benefits of participation. Moreover, the research team notified participants that they have the choice to withdraw from the study at any point. Four focus groups, one for each province (Ontario, British Columbia, Alberta, and Quebec), were conducted, with four to seven participants in each group. Focus groups were held virtually over Zoom and lasted approximately 45 minutes.* |
| 2. They have collected information only from Black Canadians and interpreted the results as they are discriminated and have less access to health care services. It would be better to compare with other privileged groups for this kind of inference. So, I would suggest more comparisons in the discussion section will enhance the power of the study. | - Thank you for your comment. We have included the following sentences to the discussion section to enhance the interpretation of our data: - *Even after controlling for socio-demographic differences (e.g., education and employment status, income) in their study sample, Gerretsen and colleagues identified higher vaccine hesitancy among Black, Indigenous, and Latinx populations in the US and Canada compared to White individuals [28] This highlights the influence of historical and contemporary systemic issues contributing to mistrust in medical interventions among racial minorities in North America, such as the unethical Tuskegee Syphilis Study in the U.S. among Black American men, and the Qu’Appelle Vaccine trial among First Nations children in Saskatchewan [28–30].* - *This indicates that, while a notable lack of willingness to receive COVID-19 vaccines was observed across various racial groups, Black Canadians exhibited the lowest inclination among all racial groups. This disparity could pose a more significant challenge within these communities, as lower willingness to receive available vaccines increases the likelihood of COVID-19’s impacts, as observed in our findings. However, this lower willingness to receive vaccines among Black communities in Canada reflect a myriad of reasons that are unique to their experiences as racialized minorities, including lower trust in vaccines and the healthcare system overall, lower health literacy, underrepresentation in vaccine trials, and experiences with discrimination and systemic racism [26]* - *We have also added more context to some of our discussion, which includes comparison groups that were not previously identified (e.g., compared to Canadians on average)* |
