# Supplementary material for: Resilience throughout COVID-19: Unmasking the realities of COVID-19 and vaccination facilitators, barriers, and attitudes among Black Canadians
Source: PLoS One. 2024 Aug 20;19(8):e0304904. doi: 10.1371/journal.pone.0304904 (PMC11335130; doi:10.1371/journal.pone.0304904)
Supplement: Attachment — Submitted filename: Response to Reviewers_OE.docx [file pone.0304904.s004.docx]

**Response to Reviewers:**

1. Lines 120 – 121 – “Interviews were conducted virtually over Zoom and lasted approximately 45 minutes”. Were incentives provided for participation?

- Thank you for your inquiry. Yes, participants were provided incentives for their participation. We have added the following sentence: C*ompensation of $30 was offered to participants upon completion of the semi-structured interviews and focus groups.*

2. Lines 128-129 – “ Focus groups were held virtually over Zoom and lasted approximately 45 minutes”. If both the semi-structured interview and focus groups were 45 minutes, how did they differ? Where the same questions used for each? Was an interview guide used?

- We have defined this distinction further in the manuscript: *The interviews were conducted first, using interview guides, and took place virtually over Zoom, each lasting approximately 45 minutes.* In the next paragraph related to focus groups, we added the following sentences: *After the completion of interviews, participants who also agreed to take part in focus groups were provided with the consent form, which were reviewed and signed. The interview guide questions were utilized to conduct the focus groups, which were held virtually via Zoom and lasted around 45 minutes.*

3. Line 135 – “they met 20 times” – very thorough research.

- Thank you for your comment.

4. Lines 168-170 – “For this study’s purposes, we limited our scope of analysis to sex (female or male) rather than gender, which encompasses a wide range of expressions of identities beyond biological attributes”. Good recognition. This could be seen as a limitation.

- Thank you for your line of thought. We have added this recognition in our limitations section: *Additionally, because we solely examined sex-related factors, our findings may not fully account for gender-related nuances, which encompass a diverse range of identities beyond biological distinctions.*

5. Line 482 – “Experiences with COVID-19 vaccines, with barriers such as policy & laws and limited access”. Consider removing the ampersand.

- Thank you for your consideration. We cannot remove the ampersand as that is a CFIR construct. Instead, we have modified the sentence to explicitly state this: *The study also highlighted the challenges concerning attitudes toward and experiences with COVID-19 vaccines, with barriers associated with CFIR constructs such as policy & laws and limited access, while knowledge and beliefs played dual roles as barriers and facilitators.*
